# Supplementary material for: Piezo2 expressed in proprioceptive neurons is essential for skeletal integrity
Source: Nat Commun. 2020 Jun 23;11:3168. doi: 10.1038/s41467-020-16971-6 (PMC7311488; doi:10.1038/s41467-020-16971-6)
Supplement: Supplementary file 1 — Supplementary Information file [file 41467_2020_16971_MOESM1_ESM.pdf]

Piezo2 Expressed in Proprioceptive Neurons is Essential for Skeletal Integrity

Assaraf et al.

Supplementary Figures

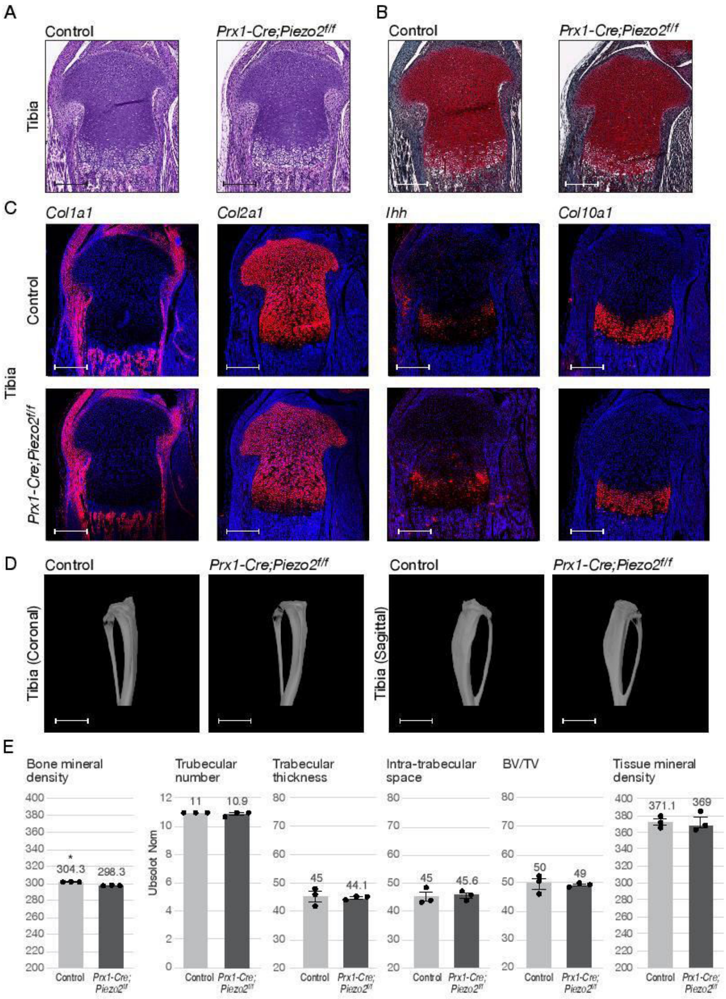

Supplementary Fig. 1 Expression of *Piezo2* in mesenchymal tissue is dispensable for limb morphogenesis.
(A) Histological sections of proximal tibia from *Prx1-Cre;Piezo2*$^{f/f}$ and control mice at P10 stained with H&E. Data are from three independent experiments.
(B) Histological sections of proximal tibia from *Prx1-Cre;Piezo2*$^{f/f}$ and control mice at P10 stained with safranin O. The data are from three independent experiments.
(C) In situ hybridization for *Col1a1*, *Col2a1*, *Ihh* and *Col10a1* on sections through the proximal tibia of *Prx1-Cre;Piezo2*$^{f/f}$ and control mice at P10. Data are from three independent experiments.
(D) 3D reconstructions of ex vivo CT scans of proximal tibia from *Prx1-Cre;Piezo2*$^{f/f}$ and control mice at P120 show similar morphologies at both coronal (left) and sagittal (right) views.
(E) Graphs comparing values of various bone density parameters between *Prx1-Cre;Piezo2*$^{f/f}$ (n=3) and control mice (n=3) at P60. Statistical significance as determined by Welch's two-sample *t*-test (from left to right): p=0.012; p=0.42; p=0.78; p=0.83; p=0.84; p=0.90. Bar and whiskers represent mean value and SEM. Source data are provided as a Source Data file.
Scale bars: 260 μm in (A-C), 6.2 mm in (D).

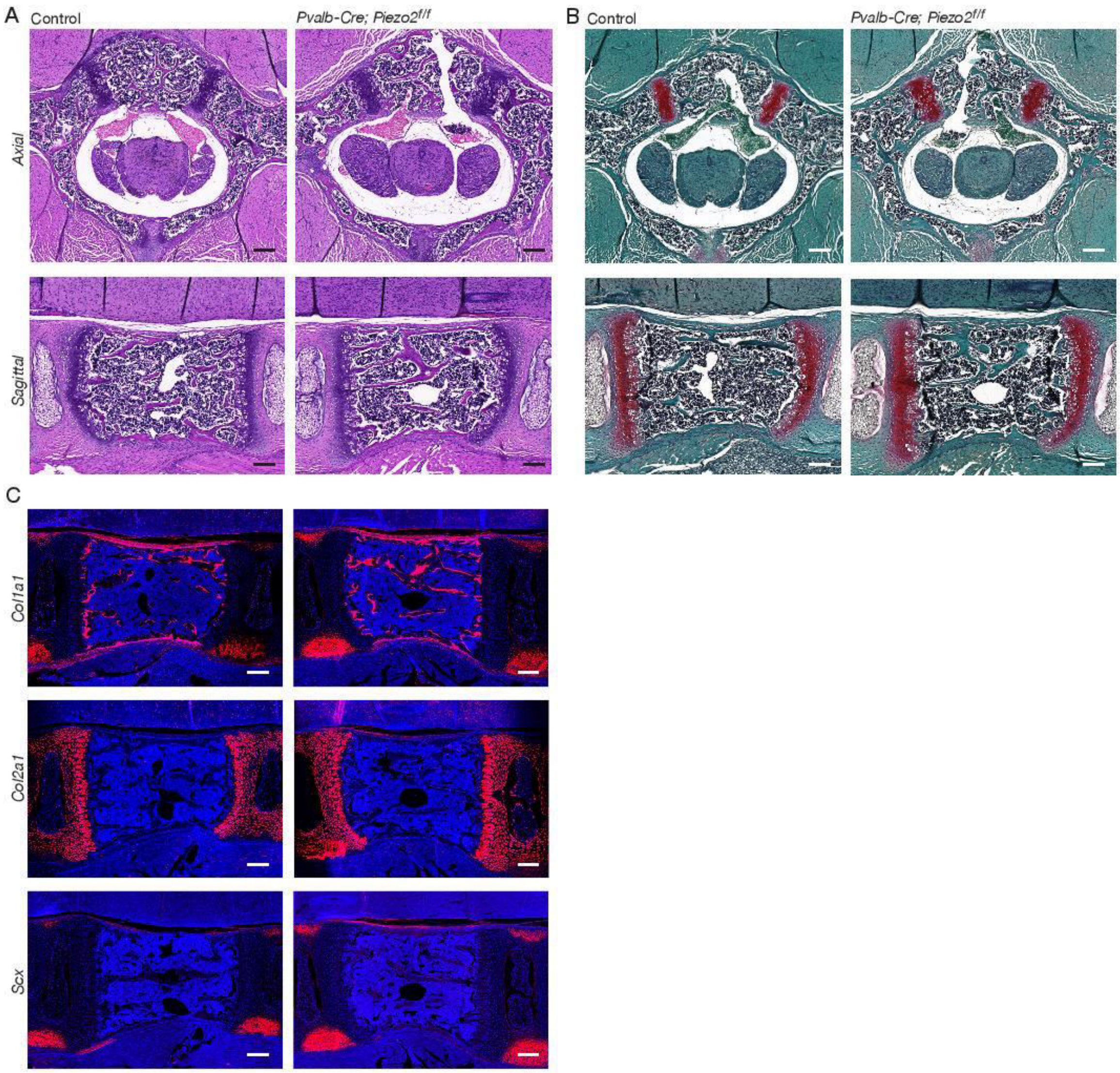

Supplementary Fig. 2. Loss of *Piezo2* in proprioceptive neurons did not result in major abnormalities in vertebrae and surrounding tissues.

(A) Safranin O-stained histological sections of vertebra from of *Pvalb-Cre; Piezo2$^{f/f}$* and control mice at P60. Data are from three independent experiments.

(B) H&E-stained histological sections of vertebra from of *Pvalb-Cre; Piezo2$^{f/f}$* and control mice at P60. Data are from three independent experiments.

(C) In situ hybridization for bone (*Col1a1*), cartilage (*Col2a1*) and tendon (*Scx*) markers in sections through the vertebra of *Pvalb-Cre; Piezo2$^{f/f}$* and control mice at P60. Data are from three independent experiments.

Scale bars: 170 μm in (A, top), 150 μm in (A, bottom), 170 μm in (B, top), 150 μm in (B, bottom), 150 μm in (C).

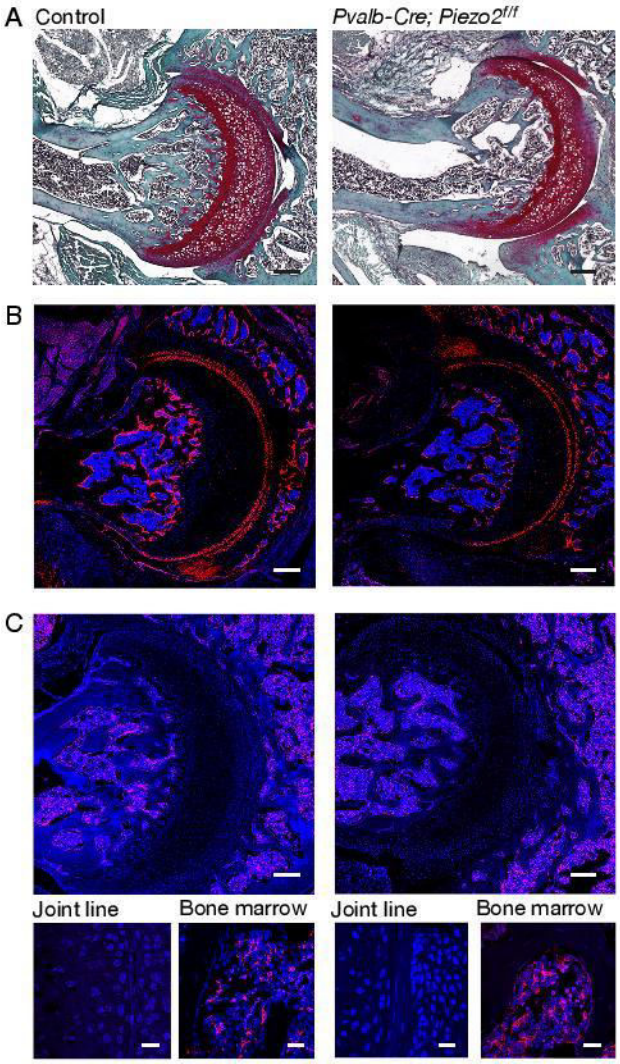

Supplementary Fig. 3. No sign for joint wearing or inflammation in dysplastic hip joints of *Pvalb-Cre; Piezo2$^{f/f}$* mice.

(A) Safranin O-stained histological sections of *Pvalb-Cre; Piezo2$^{f/f}$* and control mice at P60 show hip dysplasia and femoral cam but with no signs of wearing or inflammation in the cartilage or ligament. Data are from three independent experiments.

(B) In situ hybridization for the articular marker lubricin at the joint line of *Pvalb-Cre; Piezo2$^{f/f}$* and control mice at P60. Data are from three independent experiments.
(C) Immunofluorescence staining for the macrophage marker F4-80 on histological sections from *Pvalb-Cre; Piezo2$^{f/f}$* and control mice at P60. Data are from three independent experiments.

Scale bars: 165 μm in (A and B), 100 μm in (C, top), 25 μm in (C, bottom).

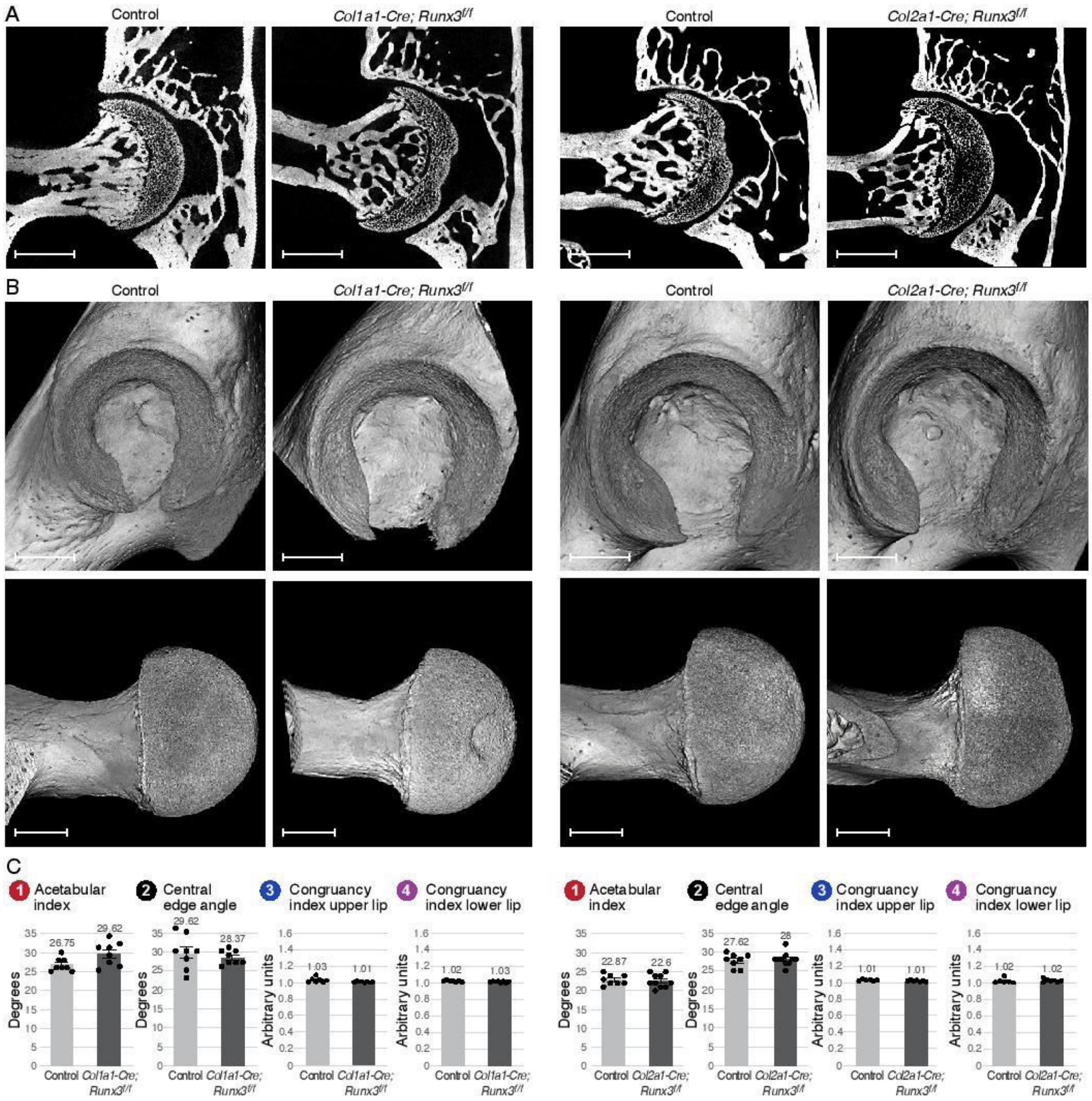

Supplementary Fig. 4. Ablation of *Runx3* from osteogenic and chondrogenic cells did not result in hip dysplasia.

(A) Ex vivo CT scans of P60 control and *Col1a1-Cre; Runx3*$^{f/f}$ mice (left panels, n = 8 in both groups) and control (n=8) and *Col2a1-Cre; Runx3*$^{f/f}$ mice (n = 10, right panels) show no signs of hip dysplasia.

(B) 3D reconstruction of ex vivo CT scans at P60 show no femoral cam or acetabular dysplasia in the *Runx3* cKO mice. Data are from three independent experiments.

(C) Graphs showing non-significant differences in index values for control and cKO mice. For *Col1a1-Cre; Runx3*$^{f/f}$ mice (left): in graphs 1,2 n = 8 in both groups, and in graphs 3,4 $n_{Control}$=7, n_{cKO}=8). For *Col2a1-Cre; Runx3*$^{f/f}$ mice (right): panels, in graphs 1,2 $n_{Control}$=8 and n_{cKO}=10, and in graphs 3,4 $n_{Control}$=6 and n_{cKO}=7). Data are presented as mean ± SEM. Source data are provided as a Source Data file. Scale bars: 220 µm in (A), 765 µm in (B, top) and 770 µm in (B, bottom).

Supplementary Tables

Supplementary Table 1. List of RNA probes used for fluorescent *in situ* hybridization.

Probe name	Genomic position	Ref-seq template	Size
Col1a1	4295 to 4475	NM_007742.4	180 bp
Col2a1	4474 to 4879	NM_001113515.2	406 bp
Col10a1	1757 to 2405	NM_009925.4	648 bp
Scx	274 to 1129	NM_198885.3	855 bp
Ihh	1560 to 2476	NM_010544.3	916 bp
Lubricin	2461 to 3011	NM_021400.3	550 bp

Supplementary Table 2. List of primers used for animal genotyping.

Name of primer	Sequence
Pvalb-Cre	F - CCTGGAAAATGCTTCTGTCCGTTTGCC<br>R - GAGTTGATAGCTGGCTGGTGGCAGATG
Prx1-Cre	F - CCTGGAAAATGCTTCTGTCCGTTTGCC<br>R - GAGTTGATAGCTGGCTGGTGGCAGATG
Col1a-Cre	F - CCTGGAAAATGCTTCTGTCCGTTTGCC<br>R - GAGTTGATAGCTGGCTGGTGGCAGATG
Col2a-Cre	F - CCTGGAAAATGCTTCTGTCCGTTTGCC<br>R - GAGTTGATAGCTGGCTGGTGGCAGATG
Wnt1-Cre	F - CCTGGAAAATGCTTCTGTCCGTTTGCC<br>R - GAGTTGATAGCTGGCTGGTGGCAGATG
Piezo2$^{loxP/loxP}$	WT FW - ACTTAGATGGGGCAGGTGCT<br>WT REV - ACTTCCCTACCCACCCATTC<br>MUT FW - ATCTACCACGGGGCTCTCTC<br>MUT REV - GCCGCTCTAGAACTAGTGGA
Egr3 KO	EGR WT FW - TGC CCC AAC CGC CGC TTA CTC TCA<br>EGR WT REV - GGC GCA CCC CCT TTC TCC GAC TTC<br>NEO REV - CGG AAC ACG GCG GCA TCA GAG
Runx3 KO	157FW - GCAAGATGGGCGAGAACAG<br>157REV - AGCACGGAGCAGAGGAAGT<br>NEO - TCTGTGACCCATGGCGATGCC
Runx3$^{loxP/loxP}$	Flox:<br>88 – GGGAGAGAGGCTGGGATGCC<br>NEO - TCTGTGACCCATGGCGATGCC<br>WT:<br>88 - GGGAGAGAGGCTGGGATGCC<br>87 –GATTCTGGAGGCTAGGAGCTC
