# Supplementary material for: Piezo2 expressed in proprioceptive neurons is essential for skeletal integrity
Source: Nat Commun. 2020 Jun 23;11:3168. doi: 10.1038/s41467-020-16971-6 (PMC7311488; doi:10.1038/s41467-020-16971-6)
Supplement: Supplementary file 2 — Reporting Summary [file 41467_2020_16971_MOESM2_ESM.pdf]

nature research

NCOMMS-19-26689B

Corresponding author(s): Eran Assaraf, Ronen Blecher, Lia Yerushalmi Heinemann, Sharon Krief, Ron Carmel Vinestock, Inbal E. Biton, Vlad Brumfeld, Ron Rotkopf, Erez Avisar, Gabriel Agar, Elazar Zelzer.

Last updated by author(s): Apr 27, 2020

Reporting Summary

Nature Research wishes to improve the reproducibility of the work that we publish. This form provides structure for consistency and transparency in reporting. For further information on Nature Research policies, see Authors & Referees and the Editorial Policy Checklist .

Statistics

For all statistical analyses, confirm that the following items are present in the figure legend, table legend, main text, or Methods section.

n/a	Confirmed	
☐	☒	The exact sample size (*n*) for each experimental group/condition, given as a discrete number and unit of measurement
☐	☒	A statement on whether measurements were taken from distinct samples or whether the same sample was measured repeatedly
☐	☒	The statistical test(s) used AND whether they are one- or two-sided<br>*Only common tests should be described solely by name; describe more complex techniques in the Methods section.*
☐	☒	A description of all covariates tested
☐	☒	A description of any assumptions or corrections, such as tests of normality and adjustment for multiple comparisons
☐	☒	A full description of the statistical parameters including central tendency (e.g. means) or other basic estimates (e.g. regression coefficient) AND variation (e.g. standard deviation) or associated estimates of uncertainty (e.g. confidence intervals)
☐	☒	For null hypothesis testing, the test statistic (e.g. *F*, *t*, *r*) with confidence intervals, effect sizes, degrees of freedom and *P* value noted<br>*Give P values as exact values whenever suitable.*
☒	☐	For Bayesian analysis, information on the choice of priors and Markov chain Monte Carlo settings
☒	☐	For hierarchical and complex designs, identification of the appropriate level for tests and full reporting of outcomes
☒	☐	Estimates of effect sizes (e.g. Cohen's *d*, Pearson's *r*), indicating how they were calculated

Our web collection on statistics for biologists contains articles on many of the points above.

Software and code

Policy information about availability of computer code

Data collection	No software was used for data collection.
Data analysis	In vivo scans were performed using TomoScope 30S Duo scanner (CT Imaging, Germany) equipped with two source-detector systems.<br>All micro-CT scans were reconstructed with a filtered back-projection algorithm using scanner software. Then, the reconstructed datasets of each animal were merged using Image J software, 1.52a (imagej.net). 3D volume rendering images were produced using Amira software, 5.2.2 (Thermo Fisher Scientific).<br>Ex vivo micro-CT Scans were performed using an Xradia MicroXCT-400 scanner.<br>Volume reconstruction for the ex vivo micro-CT Scans was done with a proprietary Zeiss software, XMReconstructor 8.2.2720.<br>Reconstructed datasets of each animal from both in vivo and ex vivo scans were merged using MATLAB software, version R2017a.<br>Images were reoriented before measurement using Microview software, version 2.5.0 (Parallax Innovations).<br>3D models from ex vivo scans were obtained using Avizo software, version 9.4 (Thermo Fisher Scientific).

For manuscripts utilizing custom algorithms or software that are central to the research but not yet described in published literature, software must be made available to editors/reviewers. We strongly encourage code deposition in a community repository (e.g. GitHub). See the Nature Research guidelines for submitting code & software for further information.

Data

Policy information about availability of data

All manuscripts must include a data availability statement. This statement should provide the following information, where applicable:

- Accession codes, unique identifiers, or web links for publicly available datasets
- A list of figures that have associated raw data
- A description of any restrictions on data availability

The data that support the findings of this study are available from the corresponding author upon reasonable request.

Field-specific reporting

Please select the one below that is the best fit for your research. If you are not sure, read the appropriate sections before making your selection.

☒ Life sciences ☐ Behavioural & social sciences ☐ Ecological, evolutionary & environmental sciences

For a reference copy of the document with all sections, see nature.com/documents/nr-reporting-summary-flat.pdf

Life sciences study design

All studies must disclose on these points even when the disclosure is negative.

Sample size	The data in this study were collected from mice that underwent genetic manipulations using well-established genetic deleter lines. These manipulations resulted in high repetition of the described phenotypes. In general, experiments were performed in three biological repetitions, as accepted in our field and as required by the institutional ethics committee (IACUC).
Data exclusions	No data were excluded from the analysis.
Replication	In order to verify measurement replication, animals from three litters of each mouse line were analyzed. All measurements are described in the Methods section. All replications were successful.
Randomization	The data were collected from genetically manipulated animals. We collected measurements from all mutant and control animals with no exclusions and animals were allocated randomly into experimental groups. Under these terms, randomization is done by Mother Nature.
Blinding	As the phenotypes of mutant animals were visually significant in all cases, we were unable to preform blind measurements.

Reporting for specific materials, systems and methods

We require information from authors about some types of materials, experimental systems and methods used in many studies. Here, indicate whether each material, system or method listed is relevant to your study. If you are not sure if a list item applies to your research, read the appropriate section before selecting a response.

Materials & experimental systems

n/a	Involved in the study	
☐	☒	Antibodies
☒	☐	Eukaryotic cell lines
☒	☐	Palaeontology
☐	☒	Animals and other organisms
☒	☐	Human research participants
☒	☐	Clinical data

Methods

n/a	Involved in the study	
☒	☐	ChIP-seq
☒	☐	Flow cytometry
☒	☐	MRI-based neuroimaging

Antibodies

Antibodies used	anti-DIG-POD (1:300, 11207733910, Roche)<br>Anti-F4/80 antibody (1:50, ab6640, Abcam)<br>biotin anti-rat (1:100 Jackson laboratories)<br>streptavidin-Cy3 (1:100, 615-222-214, Jackson ImmunoResearch)<br>Cy3-conjugated donkey anti-rat (1:100, 712-165-153, Jackson ImmunoResearch)
Validation	Anti-F4/80 antibody: This antibody recognizes the mouse F4/80 antigen, a 160 kD glycoprotein expressed by murine macrophages. This antibody is referenced in 507 publications (https://www.abcam.com/f480-antibody-cia3-1-macrophage-marker-ab6640-references.html#top-983).<br>Relevant references:

1. Wang, Hongdong et al. “Adipose group 1 innate lymphoid cells promote adipose tissue fibrosis and diabetes in obesity.” Nature communications vol. 10,1 3254. 22 Jul. 2019. doi:10.1038/s41467-019-11270-1
2. Greenhalgh, Stephen N et al. “Loss of Integrin αvβ8 in Murine Hepatocytes Accelerates Liver Regeneration.” The American journal of pathology vol. 189,2 (2019): 258-271. doi:10.1016/j.ajpath.2018.10.007

Animals and other organisms

Policy information about studies involving animals; ARRIVE guidelines recommended for reporting animal research

Laboratory animals	The generation of Runx3-null (KO; ICR background) , Egr3-null (KO; C57BL/6 background) , loxP-flanked (floxed) Runx3 (Runx3loxP/loxP), Col1a1-Cre, Col2a1-Cre, Wnt1-Cre , Pvalb-Cre, and floxed Piezo2 (Piezo2loxP/loxP) mice have been described previously. Col1a1-Runx3, Col2a1-Runx3, and Wnt1-Runx3 cKO mutants were generated by crossing males bearing the relevant Cre and a single Runx3-floxed allele (loxP/+) with a female homozygous for the Runx3 loxP allele. Pvalb-Piezo2, Col1a1-Piezo2, Col2a1-Piezo2, and Prx1-Piezo2 cKO mutants were generated by crossing males bearing the relevant Cre and a single Piezo2-floxed allele (loxP/+) with a female homozygous for the Piezo2 loxP allele. In each strain, animals lacking Cre (loxP/loxP or loxP/+) served as a control.<br>We used animals of both sexes.<br>For the measurement of spine alignment, Col1a1-Cre, Col2a1-Cre; Piezo2 cKO were scanned at P60 and P90 and Pvalb-Cre; Piezo2 cKO were scanned at P40, P60 and P90.<br>For the evaluation of hip dysplasia, animals were sacrificed at P60. For histological analysis, Pvalb-Cre; Piezo2 cKO mice were sacrificed at ages P7, P14, P45.<br>Housing conditions: Animal housing condition were supervised daily by the first author and the animal service stuff at the WIS. Animals cages included up to five animals held at a temperature of 22C +/- one degree and humidity of 50% +/- 10% with continuous oxygen supply, daily supply of food and water and weekly cage replacement.
Wild animals	The study did not involve wild animals.
Field-collected samples	The study did not involve samples collected in the field.
Ethics oversight	All experiments involving mice were approved by the Institutional Animal Care and Use Committee (IACUC) of the Weizmann Institute.

Note that full information on the approval of the study protocol must also be provided in the manuscript.
